# Draft Genome Sequence of Vibrio ostreicida Strain PP-203, the Type Strain of a Pathogen That Infects Bivalve Larvae

**DOI:** 10.1128/MRA.00913-20

**Published:** 2020-08-27

**Authors:** Ritikaa S. Kumar, Fabian Galvis, Benjamin J. Wasson, Jimmy H. Saw, David K. Oline, Juan L. Barja, Blake Ushijima, Marc J. Koyack, Susana Prado, Patrick Videau

**Affiliations:** a Department of Biology, Southern Oregon University, Ashland, Oregon, USA; b Departamento de Microbiología y Parasitología, CIBUS-Facultad de Biología, Universidad de Santiago de Compostela, Santiago de Compostela, A Coruña, Spain; c Department of Biological Sciences, The George Washington University, Washington, DC, USA; d Smithsonian Marine Station, Fort Pierce, Florida, USA; e Department of Chemistry, Southern Oregon University, Ashland, Oregon, USA; Broad Institute

## Abstract

Vibrio ostreicida is a Gram-negative gammaproteobacterium that has been shown to cause disease in bivalve larvae. Presented here is the draft genome of the type strain Vibrio ostreicida strain PP-203, which was isolated from the inner surface of an Ostrea edulis (European flat oyster) spat container with recorded deaths at a hatchery in Galicia, Spain.

## ANNOUNCEMENT

Bivalve aquaculture is a growing industry, with production increasing from 9 million tonnes at the beginning of the century to more than 16 million tonnes in 2018 (1, 2). Due to the depletion of natural beds and overexploitation, the increasing scarcity of wild seed makes hatcheries the only viable alternative to meet growing demands. Bacterial pathogens from the genus *Vibrio* have been linked to acute mortality events in hatcheries worldwide, which can result in the loss of entire batches at these facilities (3, 4). Isolated from the inner surface of Ostrea edulis (European flat oyster) spat containers in a nursery in Galicia, Spain, the Vibrio ostreicida type strain PP-203 was found to cause death to O. edulis larvae (5, 6). Sequencing the genome of the pathogenic type strain of Vibrio ostreicida will facilitate virulence factor determination and allow comparison with Vibrio ostreicida UCD-KL16, which was isolated from a seagrass leaf (Zostera marina) (7).

Vibrio ostreicida strain PP-203 was maintained in the laboratory collection of the Department of Microbiology at Universidad de Santiago de Compostela or acquired from the Leibniz Institute DSMZ (DSM 21433). Genomic DNA was extracted from cultures of V. ostreicida that had been grown overnight at 25°C, with shaking, in either Trypticase soy broth with 1.5% NaCl or lysogeny broth with 3% NaCl, using an Invitrogen Easy-DNA genomic DNA purification kit or standard phenol-chloroform extraction, respectively (8). Sequencing was performed by both the Microbial Genome Sequencing Center, LLC (Pittsburgh, PA), using 151-bp paired-end libraries prepared with the Illumina Nextera kit and run on an Illumina NextSeq 550 system, and the FISABIO Public Health Sequencing and Bioinformatics Service (Valencia, Spain), using 300-bp paired-end libraries prepared with the Illumina Nextera XT kit and run on an Illumina MiSeq system, which yielded 6,561,693 and 1,031,174 pairs of raw reads, respectively. Read quality was assessed with FastQC (http://www.bioinformatics.babraham.ac.uk/projects/fastqc), and read quality trimming and adapter sequence removal were performed using BBDuk in the BBMap package (http://sourceforge.net/projects/bbmap) with the following parameters: ktrim=r ordered minlen=50 mink=11 rcomp=f k=21 ow=t ftm=5 zl=4 qtrim=rl trimq=20 (9). Trimmed reads from both sequencing runs were assembled together with SPAdes v. 3.14.0 using the --careful option and specifying kmers of 21, 33, 55, 77, 99, and 121 (10).

This assembly produced 33 scaffolds and 37 contigs with a mean coverage of 180× and an *N*_50_ value of 213,925 bp. The draft genome totals 4,371,716 bases with a G+C content of 45.63%, and 94.2% of the genome is in scaffolds of >50 kb. Preliminary genome annotation using the Prokaryotic Genome Annotation Pipeline (PGAP) identified 4,011 genes and 88 RNAs, 76 of which are tRNAs (11). Analysis with the Rapid Annotations using Subsystems Technology (RAST) server indicated that, of the total genes, 1,172 nonhypothetical genes and 65 hypothetical genes (30% of the total) were categorized into 343 subsystems (12).

### Data availability.

This whole-genome shotgun project has been deposited in DDBJ/ENA/GenBank under the accession number JABEYA000000000. The version described in this paper is version JABEYA000000000.2. Both sets of raw sequence reads were deposited in the SRA under accession number SRR11808540 and are associated with BioSample SAMN14842399.
